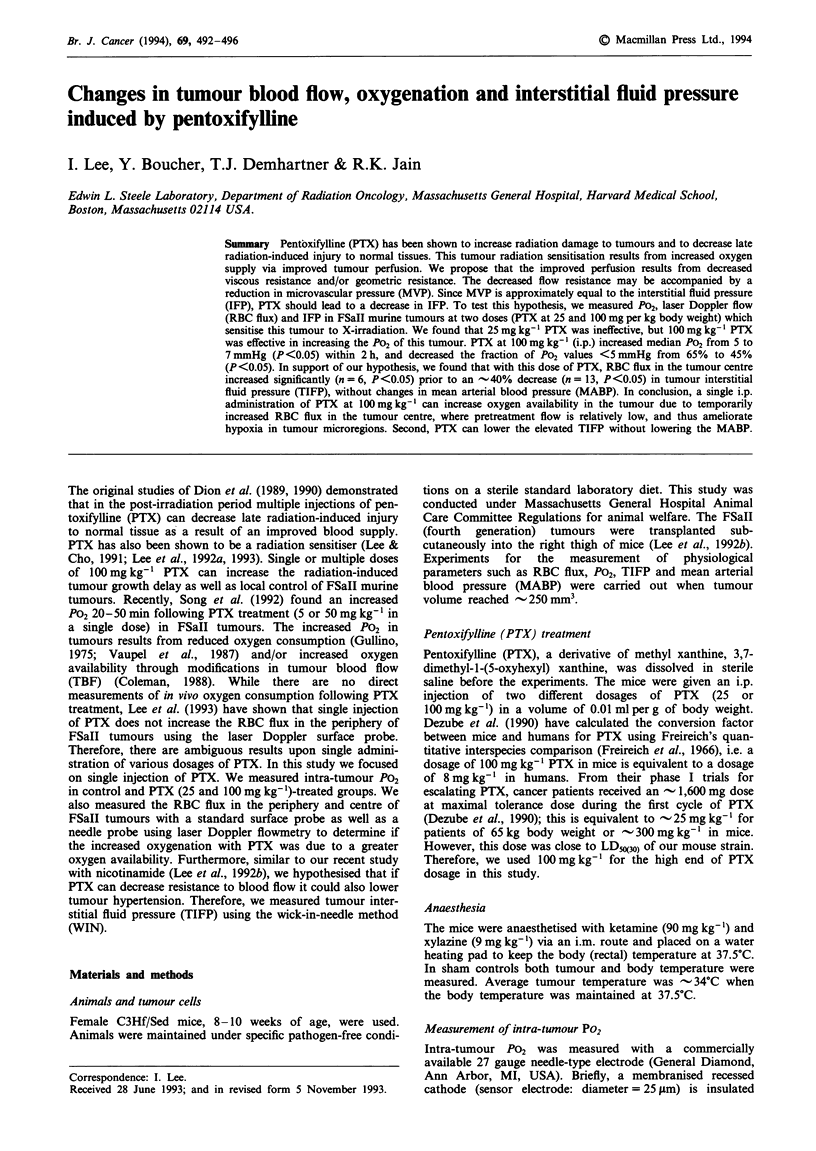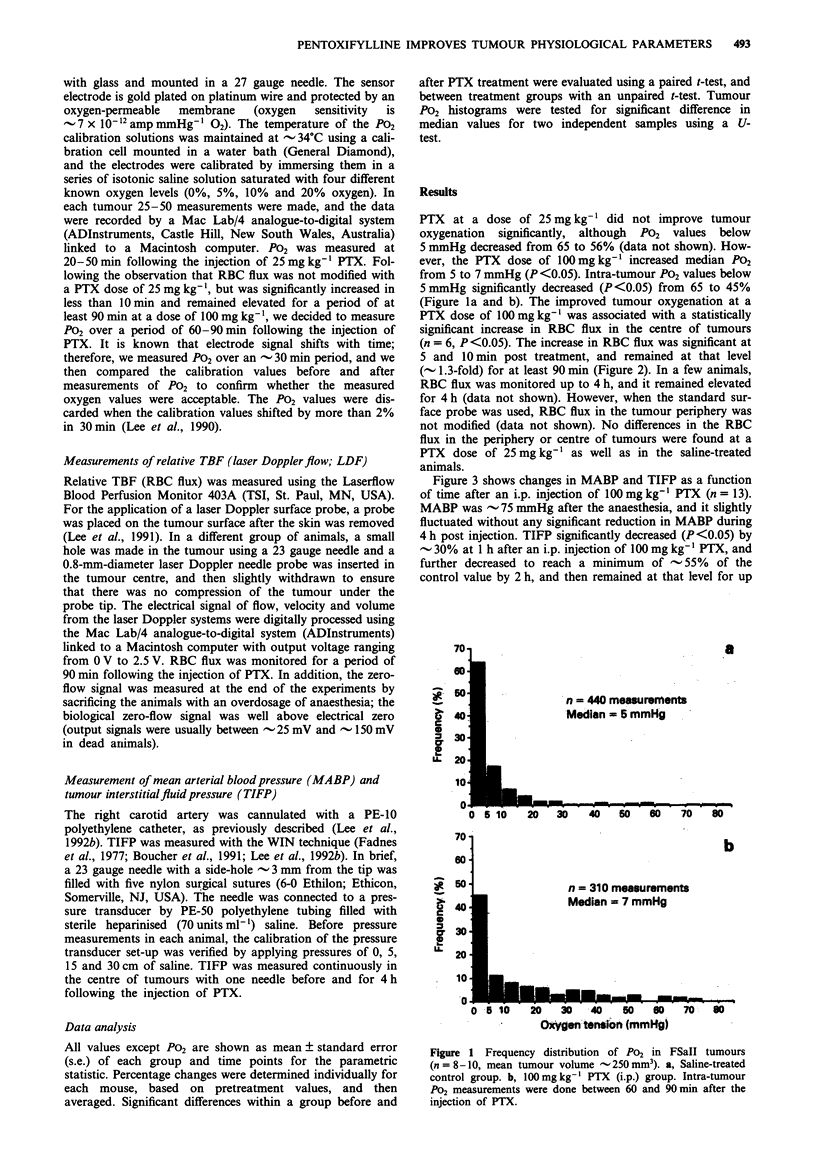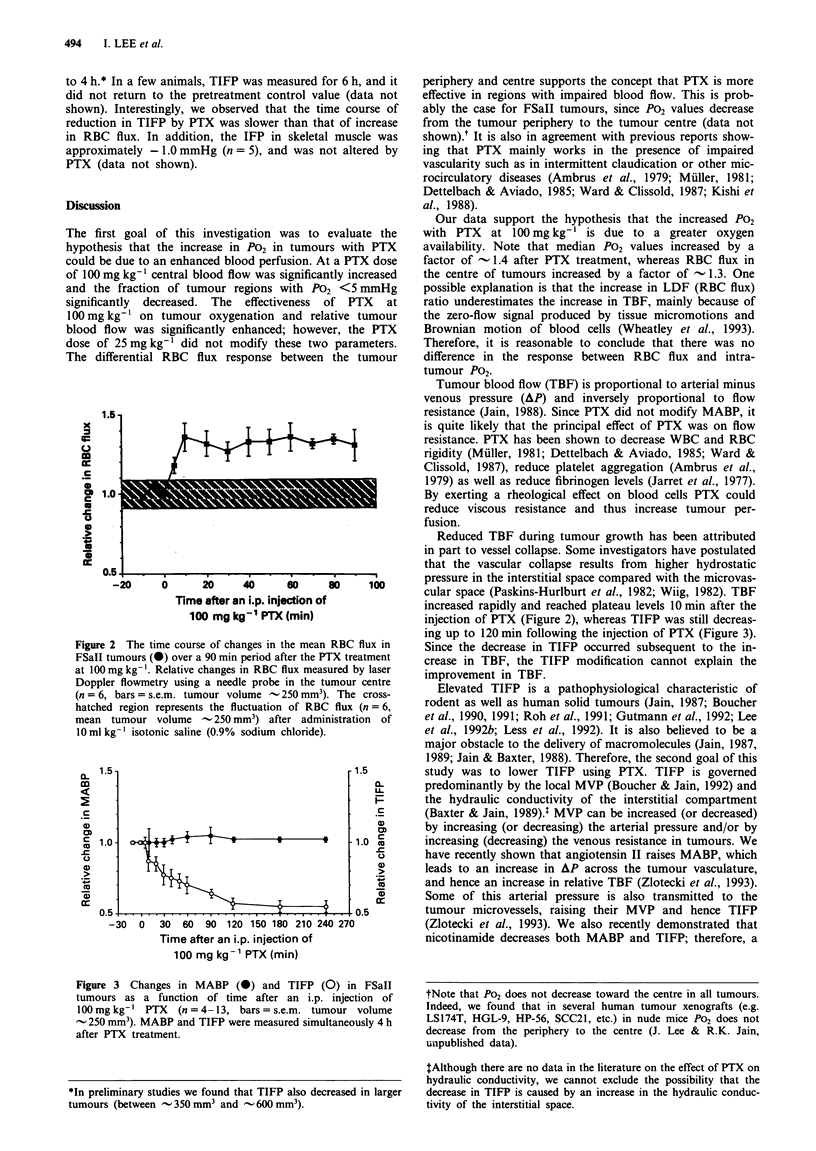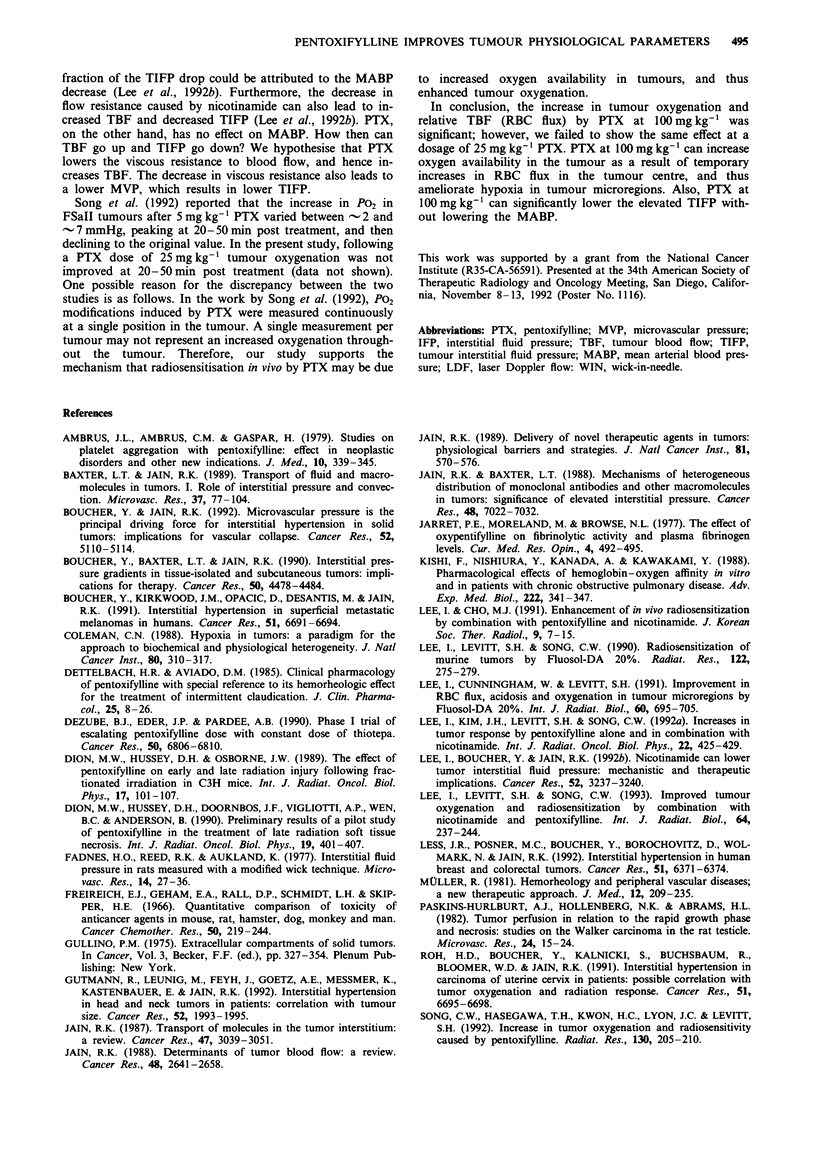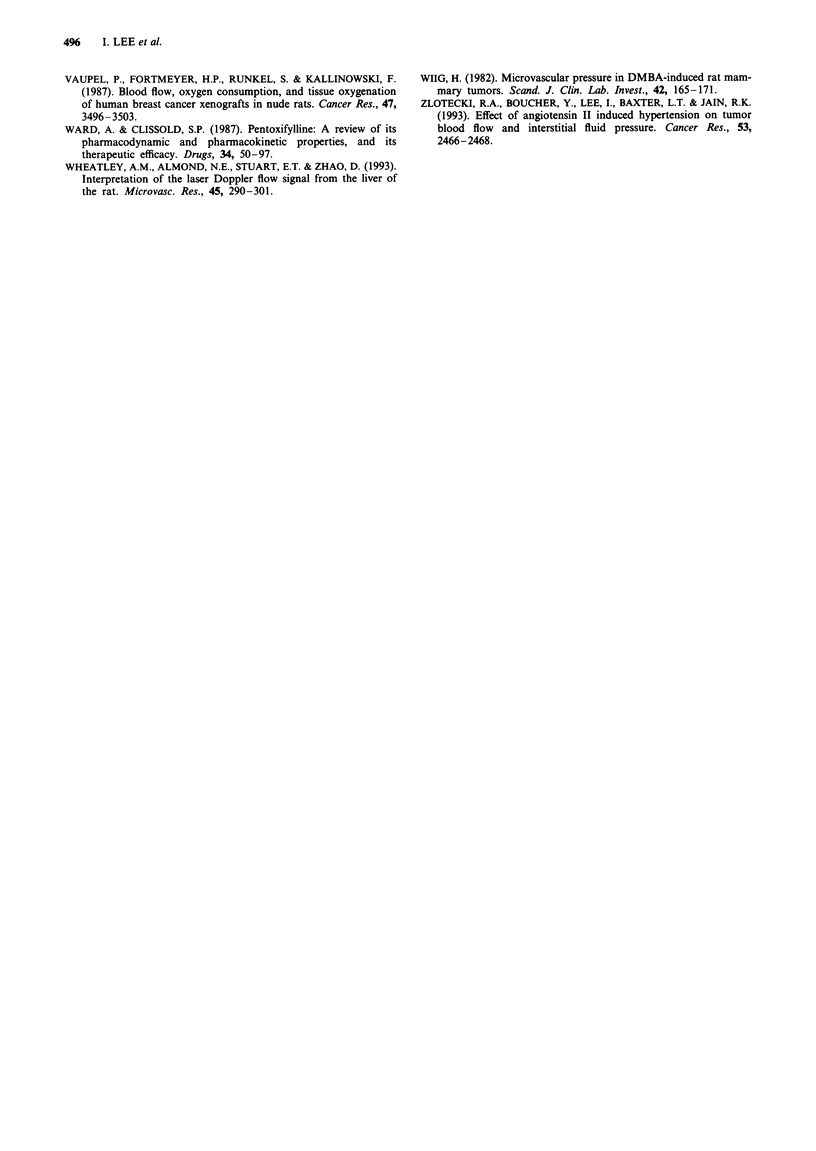# Changes in tumour blood flow, oxygenation and interstitial fluid pressure induced by pentoxifylline.

**DOI:** 10.1038/bjc.1994.89

**Published:** 1994-03

**Authors:** I. Lee, Y. Boucher, T. J. Demhartner, R. K. Jain

**Affiliations:** Edwin L. Steele Laboratory, Department of Radiation Oncology, Massachusetts General Hospital, Harvard Medical School, Boston 02114.

## Abstract

Pentoxifylline (PTX) has been shown to increase radiation damage to tumours and to decrease late radiation-induced injury to normal tissues. This tumour radiation sensitisation results from increased oxygen supply via improved tumour perfusion. We propose that the improved perfusion results from decreased viscous resistance and/or geometric resistance. The decreased flow resistance may be accompanied by a reduction in microvascular pressure (MVP). Since MVP is approximately equal to the interstitial fluid pressure (IFP), PTX should lead to a decrease in IFP. To test this hypothesis, we measured PO2, laser Doppler flow (RBC flux) and IFP in FSaII murine tumours at two doses (PTX at 25 and 100 mg per kg body weight) which sensitise this tumour to X-irradiation. We found that 25 mg kg-1 PTX was ineffective, but 100 mg kg-1 PTX was effective in increasing the PO2 of this tumour. PTX at 100 mg kg-1 (i.p.) increased median PO2 from 5 to 7 mmHg (P < 0.05) within 2 h, and decreased the fraction of PO2 values < 5 mmHg from 65% to 45% (P < 0.05). In support of our hypothesis, we found that with this dose of PTX, RBC flux in the tumour centre increased significantly (n = 6, P < 0.05) prior to an approximately 40% decrease (n = 13, P < 0.05) in tumour interstitial fluid pressure (TIFP), without changes in mean arterial blood pressure (MABP). In conclusion, a single i.p. administration of PTX at 100 mg kg-1 can increase oxygen availability in the tumour due to ameliorate hypoxia in tumour microregions. Second, PTX can lower the elevated TIFP without lowering the MABP.


Br. J. Cancer (1994), 69, 492-496 © Macmillan Press Ltd., 1994

### Changes in tumour blood flow, oxygenation and interstitial fluid pressure induced by pentoxifylline

I. Lee, Y. Boucher, T.J. Demhartner & R.K. Jain

*Edwin L. Steele Laboratory, Department of Radiation Oncology, Massachusetts General Hospital, Harvard Medical School, Boston, Massachusetts 02114 USA.*

**Summary** Pentoxifylline (PTX) has been shown to increase radiation damage to tumours and to decrease late radiation-induced injury to normal tissues. This tumour radiation sensitisation results from increased oxygen supply via improved tumour perfusion. We propose that the improved perfusion results from decreased viscous resistance and/or geometric resistance. The decreased flow resistance may be accompanied by a reduction in microvascular pressure (MVP). Since MVP is approximately equal to the interstitial fluid pressure (IFP), PTX should lead to a decrease in IFP. To test this hypothesis, we measured $Po_2$, laser Doppler flow (RBC flux) and IFP in FSaII murine tumours at two doses (PTX at 25 and 100 mg per kg body weight) which sensitise this tumour to X-irradiation. We found that 25 mg $kg^{-1}$ PTX was ineffective, but 100 mg $kg^{-1}$ PTX was effective in increasing the $Po_2$ of this tumour. PTX at 100 mg $kg^{-1}$ (i.p.) increased median $Po_2$ from 5 to 7 mmHg ($P<0.05$) within 2 h, and decreased the fraction of $Po_2$ values <5 mmHg from 65% to 45% ($P<0.05$). In support of our hypothesis, we found that with this dose of PTX, RBC flux in the tumour centre increased significantly ($n = 6$, $P<0.05$) prior to an ~40% decrease ($n = 13$, $P<0.05$) in tumour interstitial fluid pressure (TIFP), without changes in mean arterial blood pressure (MABP). In conclusion, a single i.p. administration of PTX at 100 mg $kg^{-1}$ can increase oxygen availability in the tumour due to temporarily increased RBC flux in the tumour centre, where pretreatment flow is relatively low, and thus ameliorate hypoxia in tumour microregions. Second, PTX can lower the elevated TIFP without lowering the MABP.

The original studies of Dion *et al.* (1989, 1990) demonstrated that in the post-irradiation period multiple injections of pentoxifylline (PTX) can decrease late radiation-induced injury to normal tissue as a result of an improved blood supply. PTX has also been shown to be a radiation sensitiser (Lee & Cho, 1991; Lee *et al.*, 1992*a*, 1993). Single or multiple doses of 100 mg $kg^{-1}$ PTX can increase the radiation-induced tumour growth delay as well as local control of FSaII murine tumours. Recently, Song *et al.* (1992) found an increased $Po_2$ 20-50 min following PTX treatment (5 or 50 mg $kg^{-1}$ in a single dose) in FSaII tumours. The increased $Po_2$ in tumours results from reduced oxygen consumption (Gullino, 1975; Vaupel *et al.*, 1987) and/or increased oxygen availability through modifications in tumour blood flow (TBF) (Coleman, 1988). While there are no direct measurements of *in vivo* oxygen consumption following PTX treatment, Lee *et al.* (1993) have shown that single injection of PTX does not increase the RBC flux in the periphery of FSaII tumours using the laser Doppler surface probe. Therefore, there are ambiguous results upon single administration of various dosages of PTX. In this study we focused on single injection of PTX. We measured intra-tumour $Po_2$ in control and PTX (25 and 100 mg $kg^{-1}$)-treated groups. We also measured the RBC flux in the periphery and centre of FSaII tumours with a standard surface probe as well as a needle probe using laser Doppler flowmetry to determine if the increased oxygenation with PTX was due to a greater oxygen availability. Furthermore, similar to our recent study with nicotinamide (Lee *et al.*, 1992*b*), we hypothesised that if PTX can decrease resistance to blood flow it could also lower tumour hypertension. Therefore, we measured tumour interstitial fluid pressure (TIFP) using the wick-in-needle method (WIN).

### Materials and methods

### *Animals and tumour cells*

Female C3Hf/Sed mice, 8-10 weeks of age, were used. Animals were maintained under specific pathogen-free conditions on a sterile standard laboratory diet. This study was conducted under Massachusetts General Hospital Animal Care Committee Regulations for animal welfare. The FSaII (fourth generation) tumours were transplanted subcutaneously into the right thigh of mice (Lee *et al.*, 1992*b*). Experiments for the measurement of physiological parameters such as RBC flux, $Po_2$, TIFP and mean arterial blood pressure (MABP) were carried out when tumour volume reached ~250 $mm^3$.

### *Pentoxifylline (PTX) treatment*

Pentoxifylline (PTX), a derivative of methyl xanthine, 3,7-dimethyl-1-(5-oxyhexyl) xanthine, was dissolved in sterile saline before the experiments. The mice were given an i.p. injection of two different dosages of PTX (25 or 100 mg $kg^{-1}$) in a volume of 0.01 ml per g of body weight. Dezube *et al.* (1990) have calculated the conversion factor between mice and humans for PTX using Freireich's quantitative interspecies comparison (Freireich *et al.*, 1966), i.e. a dosage of 100 mg $kg^{-1}$ PTX in mice is equivalent to a dosage of 8 mg $kg^{-1}$ in humans. From their phase I trials for escalating PTX, cancer patients received an ~1,600 mg dose at maximal tolerance dose during the first cycle of PTX (Dezube *et al.*, 1990); this is equivalent to ~25 mg $kg^{-1}$ for patients of 65 kg body weight or ~300 mg $kg^{-1}$ in mice. However, this dose was close to $LD_{50(30)}$ of our mouse strain. Therefore, we used 100 mg $kg^{-1}$ for the high end of PTX dosage in this study.

### *Anaesthesia*

The mice were anaesthetised with ketamine (90 mg $kg^{-1}$) and xylazine (9 mg $kg^{-1}$) via an i.m. route and placed on a water heating pad to keep the body (rectal) temperature at 37.5°C. In sham controls both tumour and body temperature were measured. Average tumour temperature was ~34°C when the body temperature was maintained at 37.5°C.

### *Measurement of intra-tumour* $Po_2$

Intra-tumour $Po_2$ was measured with a commercially available 27 gauge needle-type electrode (General Diamond, Ann Arbor, MI, USA). Briefly, a membranised recessed cathode (sensor electrode: diameter = 25 μm) is insulated

Correspondence: I. Lee.
Received 28 June 1993; and in revised form 5 November 1993.

with glass and mounted in a 27 gauge needle. The sensor electrode is gold plated on platinum wire and protected by an oxygen-permeable membrane (oxygen sensitivity is $\sim 7\times 10^{-12}$ amp mmHg$^{-1}$ $O_2$). The temperature of the $Po_2$ calibration solutions was maintained at ~34°C using a calibration cell mounted in a water bath (General Diamond), and the electrodes were calibrated by immersing them in a series of isotonic saline solution saturated with four different known oxygen levels (0%, 5%, 10% and 20% oxygen). In each tumour 25–50 measurements were made, and the data were recorded by a Mac Lab/4 analogue-to-digital system (ADInstruments, Castle Hill, New South Wales, Australia) linked to a Macintosh computer. $Po_2$ was measured at 20–50 min following the injection of 25 mg kg$^{-1}$ PTX. Following the observation that RBC flux was not modified with a PTX dose of 25 mg kg$^{-1}$, but was significantly increased in less than 10 min and remained elevated for a period of at least 90 min at a dose of 100 mg kg$^{-1}$, we decided to measure $Po_2$ over a period of 60–90 min following the injection of PTX. It is known that electrode signal shifts with time; therefore, we measured $Po_2$ over an ~30 min period, and we then compared the calibration values before and after measurements of $Po_2$ to confirm whether the measured oxygen values were acceptable. The $Po_2$ values were discarded when the calibration values shifted by more than 2% in 30 min (Lee *et al.*, 1990).

### *Measurements of relative TBF (laser Doppler flow; LDF)*

Relative TBF (RBC flux) was measured using the Laserflow Blood Perfusion Monitor 403A (TSI, St. Paul, MN, USA). For the application of a laser Doppler surface probe, a probe was placed on the tumour surface after the skin was removed (Lee *et al.*, 1991). In a different group of animals, a small hole was made in the tumour using a 23 gauge needle and a 0.8-mm-diameter laser Doppler needle probe was inserted in the tumour centre, and then slightly withdrawn to ensure that there was no compression of the tumour under the probe tip. The electrical signal of flow, velocity and volume from the laser Doppler systems were digitally processed using the Mac Lab/4 analogue-to-digital system (ADInstruments) linked to a Macintosh computer with output voltage ranging from 0 V to 2.5 V. RBC flux was monitored for a period of 90 min following the injection of PTX. In addition, the zero-flow signal was measured at the end of the experiments by sacrificing the animals with an overdosage of anaesthesia; the biological zero-flow signal was well above electrical zero (output signals were usually between ~25 mV and ~150 mV in dead animals).

### *Measurement of mean arterial blood pressure (MABP) and tumour interstitial fluid pressure (TIFP)*

The right carotid artery was cannulated with a PE-10 polyethylene catheter, as previously described (Lee *et al.*, 1992*b*). TIFP was measured with the WIN technique (Fadnes *et al.*, 1977; Boucher *et al.*, 1991; Lee *et al.*, 1992*b*). In brief, a 23 gauge needle with a side-hole ~3 mm from the tip was filled with five nylon surgical sutures (6-0 Ethilon; Ethicon, Somerville, NJ, USA). The needle was connected to a pressure transducer by PE-50 polyethylene tubing filled with sterile heparinised (70 units ml$^{-1}$) saline. Before pressure measurements in each animal, the calibration of the pressure transducer set-up was verified by applying pressures of 0, 5, 15 and 30 cm of saline. TIFP was measured continuously in the centre of tumours with one needle before and for 4 h following the injection of PTX.

### *Data analysis*

All values except $Po_2$ are shown as mean ± standard error (s.e.) of each group and time points for the parametric statistic. Percentage changes were determined individually for each mouse, based on pretreatment values, and then averaged. Significant differences within a group before and after PTX treatment were evaluated using a paired *t*-test, and between treatment groups with an unpaired *t*-test. Tumour $Po_2$ histograms were tested for significant difference in median values for two independent samples using a *U*-test.

### Results

PTX at a dose of 25 mg kg$^{-1}$ did not improve tumour oxygenation significantly, although $Po_2$ values below 5 mmHg decreased from 65 to 56% (data not shown). However, the PTX dose of 100 mg kg$^{-1}$ increased median $Po_2$ from 5 to 7 mmHg ($P<0.05$). Intra-tumour $Po_2$ values below 5 mmHg significantly decreased ($P<0.05$) from 65 to 45% (Figure 1a and b). The improved tumour oxygenation at a PTX dose of 100 mg kg$^{-1}$ was associated with a statistically significant increase in RBC flux in the centre of tumours ($n=6$, $P<0.05$). The increase in RBC flux was significant at 5 and 10 min post treatment, and remained at that level (~1.3-fold) for at least 90 min (Figure 2). In a few animals, RBC flux was monitored up to 4 h, and it remained elevated for 4 h (data not shown). However, when the standard surface probe was used, RBC flux in the tumour periphery was not modified (data not shown). No differences in the RBC flux in the periphery or centre of tumours were found at a PTX dose of 25 mg kg$^{-1}$ as well as in the saline-treated animals.

Figure 3 shows changes in MABP and TIFP as a function of time after an i.p. injection of 100 mg kg$^{-1}$ PTX ($n=13$). MABP was ~75 mmHg after the anaesthesia, and it slightly fluctuated without any significant reduction in MABP during 4 h post injection. TIFP significantly decreased ($P<0.05$) by ~30% at 1 h after an i.p. injection of 100 mg kg$^{-1}$ PTX, and further decreased to reach a minimum of ~55% of the control value by 2 h, and then remained at that level for up

**Figure 1** Frequency distribution of $Po_2$ in FSaII tumours ($n=8-10$, mean tumour volume ~250 mm$^3$). **a**, Saline-treated control group. **b**, 100 mg kg$^{-1}$ PTX (i.p.) group. Intra-tumour $Po_2$ measurements were done between 60 and 90 min after the injection of PTX.

to 4 h.* In a few animals, TIFP was measured for 6 h, and it did not return to the pretreatment control value (data not shown). Interestingly, we observed that the time course of reduction in TIFP by PTX was slower than that of increase in RBC flux. In addition, the IFP in skeletal muscle was approximately − 1.0 mmHg ($n = 5$), and was not altered by PTX (data not shown).

### Discussion

The first goal of this investigation was to evaluate the hypothesis that the increase in $Po_2$ in tumours with PTX could be due to an enhanced blood perfusion. At a PTX dose of 100 mg kg$^{-1}$ central blood flow was significantly increased and the fraction of tumour regions with $Po_2$ <5 mmHg significantly decreased. The effectiveness of PTX at 100 mg kg$^{-1}$ on tumour oxygenation and relative tumour blood flow was significantly enhanced; however, the PTX dose of 25 mg kg$^{-1}$ did not modify these two parameters. The differential RBC flux response between the tumour periphery and centre supports the concept that PTX is more effective in regions with impaired blood flow. This is probably the case for FSaII tumours, since $Po_2$ values decrease from the tumour periphery to the tumour centre (data not shown).† It is also in agreement with previous reports showing that PTX mainly works in the presence of impaired vascularity such as in intermittent claudication or other microrocirculatory diseases (Ambrus *et al.*, 1979; Müller, 1981; Dettelbach & Aviado, 1985; Ward & Clissold, 1987; Kishi *et al.*, 1988).

Our data support the hypothesis that the increased $Po_2$ with PTX at 100 mg kg$^{-1}$ is due to a greater oxygen availability. Note that median $Po_2$ values increased by a factor of ~1.4 after PTX treatment, whereas RBC flux in the centre of tumours increased by a factor of ~1.3. One possible explanation is that the increase in LDF (RBC flux) ratio underestimates the increase in TBF, mainly because of the zero-flow signal produced by tissue micromotions and Brownian motion of blood cells (Wheatley *et al.*, 1993). Therefore, it is reasonable to conclude that there was no difference in the response between RBC flux and intratumour $Po_2$.

Tumour blood flow (TBF) is proportional to arterial minus venous pressure ($\Delta P$) and inversely proportional to flow resistance (Jain, 1988). Since PTX did not modify MABP, it is quite likely that the principal effect of PTX was on flow resistance. PTX has been shown to decrease WBC and RBC rigidity (Müller, 1981; Dettelbach & Aviado, 1985; Ward & Clissold, 1987), reduce platelet aggregation (Ambrus *et al.*, 1979) as well as reduce fibrinogen levels (Jarret *et al.*, 1977). By exerting a rheological effect on blood cells PTX could reduce viscous resistance and thus increase tumour perfusion.

Reduced TBF during tumour growth has been attributed in part to vessel collapse. Some investigators have postulated that the vascular collapse results from higher hydrostatic pressure in the interstitial space compared with the microvascular space (Paskins-Hurlburt *et al.*, 1982; Wiig, 1982). TBF increased rapidly and reached plateau levels 10 min after the injection of PTX (Figure 2), whereas TIFP was still decreasing up to 120 min following the injection of PTX (Figure 3). Since the decrease in TIFP occurred subsequent to the increase in TBF, the TIFP modification cannot explain the improvement in TBF.

Elevated TIFP is a pathophysiological characteristic of rodent as well as human solid tumours (Jain, 1987; Boucher *et al.*, 1990, 1991; Roh *et al.*, 1991; Gutmann *et al.*, 1992; Lee *et al.*, 1992*b*; Less *et al.*, 1992). It is also believed to be a major obstacle to the delivery of macromolecules (Jain, 1987, 1989; Jain & Baxter, 1988). Therefore, the second goal of this study was to lower TIFP using PTX. TIFP is governed predominantly by the local MVP (Boucher & Jain, 1992) and the hydraulic conductivity of the interstitial compartment (Baxter & Jain, 1989).‡ MVP can be increased (or decreased) by increasing (or decreasing) the arterial pressure and/or by increasing (decreasing) the venous resistance in tumours. We have recently shown that angiotensin II raises MABP, which leads to an increase in $\Delta P$ across the tumour vasculature, and hence an increase in relative TBF (Zlotecki *et al.*, 1993). Some of this arterial pressure is also transmitted to the tumour microvessels, raising their MVP and hence TIFP (Zlotecki *et al.*, 1993). We also recently demonstrated that nicotinamide decreases both MABP and TIFP; therefore, a

**Figure 2** The time course of changes in the mean RBC flux in FSaII tumours (●) over a 90 min period after the PTX treatment at 100 mg kg$^{-1}$. Relative changes in RBC flux measured by laser Doppler flowmetry using a needle probe in the tumour centre ($n = 6$, bars = s.e.m. tumour volume ~250 mm$^3$). The cross-hatched region represents the fluctuation of RBC flux ($n = 6$, mean tumour volume ~250 mm$^3$) after administration of 10 ml kg$^{-1}$ isotonic saline (0.9% sodium chloride).

**Figure 3** Changes in MABP (●) and TIFP (○) in FSaII tumours as a function of time after an i.p. injection of 100 mg kg$^{-1}$ PTX ($n = 4-13$, bars = s.e.m. tumour volume ~250 mm$^3$). MABP and TIFP were measured simultaneously 4 h after PTX treatment.

*In preliminary studies we found that TIFP also decreased in larger tumours (between ~350 mm$^3$ and ~600 mm$^3$).

†Note that $Po_2$ does not decrease toward the centre in all tumours. Indeed, we found that in several human tumour xenografts (e.g. LS174T, HGL-9, HP-56, SCC21, etc.) in nude mice $Po_2$ does not decrease from the periphery to the centre (J. Lee & R.K. Jain, unpublished data).

‡Although there are no data in the literature on the effect of PTX on hydraulic conductivity, we cannot exclude the possibility that the decrease in TIFP is caused by an increase in the hydraulic conductivity of the interstitial space.

fraction of the TIFP drop could be attributed to the MABP decrease (Lee *et al.*, 1992*b*). Furthermore, the decrease in flow resistance caused by nicotinamide can also lead to increased TBF and decreased TIFP (Lee *et al.*, 1992*b*). PTX, on the other hand, has no effect on MABP. How then can TBF go up and TIFP go down? We hypothesise that PTX lowers the viscous resistance to blood flow, and hence increases TBF. The decrease in viscous resistance also leads to a lower MVP, which results in lower TIFP.

Song *et al.* (1992) reported that the increase in $Po_2$ in FSaII tumours after $5\,mg\,kg^{-1}$ PTX varied between $\sim 2$ and $\sim 7$ mmHg, peaking at 20–50 min post treatment, and then declining to the original value. In the present study, following a PTX dose of $25\,mg\,kg^{-1}$ tumour oxygenation was not improved at 20–50 min post treatment (data not shown). One possible reason for the discrepancy between the two studies is as follows. In the work by Song *et al.* (1992), $Po_2$ modifications induced by PTX were measured continuously at a single position in the tumour. A single measurement per tumour may not represent an increased oxygenation throughout the tumour. Therefore, our study supports the mechanism that radiosensitisation *in vivo* by PTX may be due to increased oxygen availability in tumours, and thus enhanced tumour oxygenation.

In conclusion, the increase in tumour oxygenation and relative TBF (RBC flux) by PTX at $100\,mg\,kg^{-1}$ was significant; however, we failed to show the same effect at a dosage of $25\,mg\,kg^{-1}$ PTX. PTX at $100\,mg\,kg^{-1}$ can increase oxygen availability in the tumour as a result of temporary increases in RBC flux in the tumour centre, and thus ameliorate hypoxia in tumour microregions. Also, PTX at $100\,mg\,kg^{-1}$ can significantly lower the elevated TIFP without lowering the MABP.

This work was supported by a grant from the National Cancer Institute (R35-CA-56591). Presented at the 34th American Society of Therapeutic Radiology and Oncology Meeting, San Diego, California, November 8–13, 1992 (Poster No. 1116).

**Abbreviations:** PTX, pentoxifylline; MVP, microvascular pressure; IFP, interstitial fluid pressure; TBF, tumour blood flow; TIFP, tumour interstitial fluid pressure; MABP, mean arterial blood pressure; LDF, laser Doppler flow: WIN, wick-in-needle.

### References

AMBRUS, J.L., AMBRUS, C.M. & GASPAR, H. (1979). Studies on platelet aggregation with pentoxifylline: effect in neoplastic disorders and other new indications. *J. Med.*, **10**, 339–345.

BAXTER, L.T. & JAIN, R.K. (1989). Transport of fluid and macromolecules in tumors. I. Role of interstitial pressure and convection. *Microvasc. Res.*, **37**, 77–104.

BOUCHER, Y. & JAIN, R.K. (1992). Microvascular pressure is the principal driving force for interstitial hypertension in solid tumors: implications for vascular collapse. *Cancer Res.*, **52**, 5110–5114.

BOUCHER, Y., BAXTER, L.T. & JAIN, R.K. (1990). Interstitial pressure gradients in tissue-isolated and subcutaneous tumors: implications for therapy. *Cancer Res.*, **50**, 4478–4484.

BOUCHER, Y., KIRKWOOD, J.M., OPACIC, D., DESANTIS, M. & JAIN, R.K. (1991). Interstitial hypertension in superficial metastatic melanomas in humans. *Cancer Res.*, **51**, 6691–6694.

COLEMAN, C.N. (1988). Hypoxia in tumors: a paradigm for the approach to biochemical and physiological heterogeneity. *J. Natl Cancer Inst.*, **80**, 310–317.

DETTELBACH, H.R. & AVIADO, D.M. (1985). Clinical pharmacology of pentoxifylline with special reference to its hemorheologic effect for the treatment of intermittent claudication. *J. Clin. Pharmacol.*, **25**, 8–26.

DEZUBE, B.J., EDER, J.P. & PARDEE, A.B. (1990). Phase I trial of escalating pentoxifylline dose with constant dose of thiotepa. *Cancer Res.*, **50**, 6806–6810.

DION, M.W., HUSSEY, D.H. & OSBORNE, J.W. (1989). The effect of pentoxifylline on early and late radiation injury following fractionated irradiation in C3H mice. *Int. J. Radiat. Oncol. Biol. Phys.*, **17**, 101–107.

DION, M.W., HUSSEY, D.H., DOORNBOS, J.F., VIGLIOTTI, A.P., WEN, B.C. & ANDERSON, B. (1990). Preliminary results of a pilot study of pentoxifylline in the treatment of late radiation soft tissue necrosis. *Int. J. Radiat. Oncol. Biol. Phys.*, **19**, 401–407.

FADNES, H.O., REED, R.K. & AUKLAND, K. (1977). Interstitial fluid pressure in rats measured with a modified wick technique. *Microvasc. Res.*, **14**, 27–36.

FREIREICH, E.J., GEHAM, E.A., RALL, D.P., SCHMIDT, L.H. & SKIPPER, H.E. (1966). Quantitative comparison of toxicity of anticancer agents in mouse, rat, hamster, dog, monkey and man. *Cancer Chemother. Res.*, **50**, 219–244.

GULLINO, P.M. (1975). Extracellular compartments of solid tumors. In *Cancer*, Vol. 3, Becker, F.F. (ed.), pp. 327–354. Plenum Publishing: New York.

GUTMANN, R., LEUNIG, M., FEYH, J., GOETZ, A.E., MESSMER, K., KASTENBAUER, E. & JAIN, R.K. (1992). Interstitial hypertension in head and neck tumors in patients: correlation with tumour size. *Cancer Res.*, **52**, 1993–1995.

JAIN, R.K. (1987). Transport of molecules in the tumor interstitium: a review. *Cancer Res.*, **47**, 3039–3051.

JAIN, R.K. (1988). Determinants of tumor blood flow: a review. *Cancer Res.*, **48**, 2641–2658.

JAIN, R.K. (1989). Delivery of novel therapeutic agents in tumors: physiological barriers and strategies. *J. Natl Cancer Inst.*, **81**, 570–576.

JAIN, R.K. & BAXTER, L.T. (1988). Mechanisms of heterogeneous distribution of monoclonal antibodies and other macromolecules in tumors: significance of elevated interstitial pressure. *Cancer Res.*, **48**, 7022–7032.

JARRET, P.E., MORELAND, M. & BROWSE, N.L. (1977). The effect of oxypentifylline on fibrinolytic activity and plasma fibrinogen levels. *Cur. Med. Res. Opin.*, **4**, 492–495.

KISHI, F., NISHIURA, Y., KANADA, A. & KAWAKAMI, Y. (1988). Pharmacological effects of hemoglobin–oxygen affinity *in vitro* and in patients with chronic obstructive pulmonary disease. *Adv. Exp. Med. Biol.*, **222**, 341–347.

LEE, I. & CHO, M.J. (1991). Enhancement of *in vivo* radiosensitization by combination with pentoxifylline and nicotinamide. *J. Korean Soc. Ther. Radiol.*, **9**, 7–15.

LEE, I., LEVITT, S.H. & SONG, C.W. (1990). Radiosensitization of murine tumors by Fluosol-DA 20%. *Radiat. Res.*, **122**, 275–279.

LEE, I., CUNNINGHAM, W. & LEVITT, S.H. (1991). Improvement in RBC flux, acidosis and oxygenation in tumour microregions by Fluosol-DA 20%. *Int. J. Radiat. Biol.*, **60**, 695–705.

LEE, I., KIM, J.H., LEVITT, S.H. & SONG, C.W. (1992*a*). Increases in tumor response by pentoxifylline alone and in combination with nicotinamide. *Int. J. Radiat. Oncol. Biol. Phys.*, **22**, 425–429.

LEE, I., BOUCHER, Y. & JAIN, R.K. (1992*b*). Nicotinamide can lower tumor interstitial fluid pressure: mechanistic and therapeutic implications. *Cancer Res.*, **52**, 3237–3240.

LEE, I., LEVITT, S.H. & SONG, C.W. (1993). Improved tumour oxygenation and radiosensitization by combination with nicotinamide and pentoxifylline. *Int. J. Radiat. Biol.*, **64**, 237–244.

LESS, J.R., POSNER, M.C., BOUCHER, Y., BOROCHOVITZ, D., WOLMARK, N. & JAIN, R.K. (1992). Interstitial hypertension in human breast and colorectal tumors. *Cancer Res.*, **51**, 6371–6374.

MÜLLER, R. (1981). Hemorheology and peripheral vascular diseases; a new therapeutic approach. *J. Med.*, **12**, 209–235.

PASKINS-HURLBURT, A.J., HOLLENBERG, N.K. & ABRAMS, H.L. (1982). Tumor perfusion in relation to the rapid growth phase and necrosis: studies on the Walker carcinoma in the rat testicle. *Microvasc. Res.*, **24**, 15–24.

ROH, H.D., BOUCHER, Y., KALNICKI, S., BUCHSBAUM, R., BLOOMER, W.D. & JAIN, R.K. (1991). Interstitial hypertension in carcinoma of uterine cervix in patients: possible correlation with tumor oxygenation and radiation response. *Cancer Res.*, **51**, 6695–6698.

SONG, C.W., HASEGAWA, T.H., KWON, H.C., LYON, J.C. & LEVITT, S.H. (1992). Increase in tumor oxygenation and radiosensitivity caused by pentoxifylline. *Radiat. Res.*, **130**, 205–210.

VAUPEL, P., FORTMEYER, H.P., RUNKEL, S. & KALLINOWSKI, F. (1987). Blood flow, oxygen consumption, and tissue oxygenation of human breast cancer xenografts in nude rats. *Cancer Res.*, **47**, 3496–3503.

WARD, A. & CLISSOLD, S.P. (1987). Pentoxifylline: A review of its pharmacodynamic and pharmacokinetic properties, and its therapeutic efficacy. *Drugs*, **34**, 50–97.

WHEATLEY, A.M., ALMOND, N.E., STUART, E.T. & ZHAO, D. (1993). Interpretation of the laser Doppler flow signal from the liver of the rat. *Microvasc. Res.*, **45**, 290–301.

WIIG, H. (1982). Microvascular pressure in DMBA-induced rat mammary tumors. *Scand. J. Clin. Lab. Invest.*, **42**, 165–171.

ZLOTECKI, R.A., BOUCHER, Y., LEE, I., BAXTER, L.T. & JAIN, R.K. (1993). Effect of angiotensin II induced hypertension on tumor blood flow and interstitial fluid pressure. *Cancer Res.*, **53**, 2466–2468.